# The longitudinal mental health impact of Fukushima nuclear disaster exposures and public criticism among power plant workers: the Fukushima NEWS Project study

**DOI:** 10.1017/S003329171600194X

**Published:** 2016-08-18

**Authors:** Y. Tanisho, J. Shigemura, K. Kubota, T. Tanigawa, E. J. Bromet, S. Takahashi, Y. Matsuoka, D. Nishi, M. Nagamine, N. Harada, M. Tanichi, Y. Takahashi, K. Shimizu, S. Nomura, A. Yoshino, Fukushima NEWS Project Collaborators

**Affiliations:** 1 Health and Global Policy Institute, Chiyoda-ku, Tokyo, Japan; 2 Department of Psychiatry, National Defense Medical College, Tokorozawa, Saitama, Japan; 3 Department of Biostatistics, School of Medicine, Yokohama City University, Kanazawa-ku, Yokohama, Japan; 4 Department of Public Health, School of Medicine, Juntendo University, Bunkyo-ku, Tokyo, Japan; 5 Department of Psychiatry and Behavioral Science, Stony Brook University School of Medicine, Stony Brook, NY, USA; 6 Department of Disaster Psychiatry, Faculty of Medicine, University of Tsukuba, Tsukuba, Ibaraki, Japan; 7 Department of Health Care Research, Center for Public Health Sciences, National Cancer Center, Chuo-ku, Tokyo, Japan; 8 Department of Mental Health Policy and Evaluation, National Institute of Mental Health, National Center of Neurology and Psychiatry, Kodaira, Tokyo, Japan; 9 Division of Behavioral Science, National Defense Medical College Research Institute, National Defense Medical College, Tokorozawa, Saitama, Japan; 10 Nursing Science of Community Health Care System, Department of Nursing, Tohoku University School of Health Sciences, Sendai, Japan; 11 Rokubancho Mental Clinic, Japan Depression Center, Chiyoda-ku, Tokyo, Japan

**Keywords:** Disaster mental health, Fukushima Daiichi Nuclear Power Plant disaster, general psychological distress, Great East Japan Earthquake, post-traumatic stress disorder

## Abstract

**Background:**

The Fukushima Daiichi and Daini Nuclear Power Plant workers experienced multiple stressors as both victims and onsite workers after the 2011 Great East Japan Earthquake and subsequent nuclear accidents. Previous studies found that disaster-related exposures, including discrimination/slurs, were associated with their mental health. Their long-term impact has yet to be investigated.

**Method:**

A total of 968 plant workers (Daiichi, *n* = 571; Daini, *n* = 397) completed self-written questionnaires 2–3 months (time 1) and 14–15 months (time 2) after the disaster (response rate 55.0%). Sociodemographics, disaster-related experiences, and peritraumatic distress were assessed at time 1. At time 1 and time 2, general psychological distress (GPD) and post-traumatic stress response (PTSR) were measured, respectively, using the K6 scale and Impact of Event Scale Revised. We examined multivariate covariates of time 2 GPD and PTSR, adjusting for autocorrelations in the hierarchical multiple regression analyses.

**Results:**

Higher GPD at time 2 was predicted by higher GPD at time 1 (*β* = 0.491, *p* < 0.001) and discrimination/slurs experiences at time 1 (*β* = 0.065, *p* = 0.025, adjusted *R*^2^ = 0.24). Higher PTSR at time 2 was predicted with higher PTSR at time 1 (*β* = 0.548, *p* < 0.001), higher age (*β* = 0.085, *p* = 0.005), and discrimination/slurs experiences at time 1 (*β* = 0.079, *p* = 0.003, adjusted *R*^2^ = 0.36).

**Conclusions:**

Higher GPD at time 2 was predicted by higher GPD and discrimination/slurs experience at time 1. Higher PTSR at time 2 was predicted by higher PTSR, higher age, and discrimination/slurs experience at time 1.

## Introduction

On 11 March 2011, a 9.0 magnitude earthquake and a series of subsequent tsunamis struck the northeastern coast of Japan. This disaster led to a nuclear disaster at the Tokyo Electric Power Company (TEPCO) Fukushima Daiichi Nuclear Power Plant (Daiichi). The Fukushima nuclear disaster involved a series of explosions and complete meltdowns, and proved to be the largest nuclear disaster since the 1986 Chernobyl accident. Although the Fukushima Daini Nuclear Power Plant (Daini), located 12 km south of Daiichi, also experienced damage from the tsunami, the situation did not escalate to the point of meltdown (Shigemura *et al*. 2014).

Research following Chernobyl and the 1979 Three Mile Island (TMI) nuclear power plant accidents showed that the release of radioactive materials, both real and perceived, created immense fear and uncertainty among the public, in part due to the invisible nature of radioactivity. Related to this fear, both accidents produced adverse mental health consequences that persisted for decades (Bromet *et al*. 2011). Among the workers, however, the TMI and Chernobyl studies diverged. TMI studies were conducted at multiple points within the first 4 years after the accident. Acute effects on distress were found (Kasl *et al.* 1981*a*, *b*), but no differences between TMI and comparison workers were noted later (Parkinson & Bromet, 1983). No methodologically transparent studies of Chernobyl workers (referred to as liquidators) appeared until 1997, when an excess suicide mortality rate was reported in Estonian liquidators (Rahu *et al*. 1997), a finding extended and confirmed over time (Kasl *et al.* 1981*a*, *b*). An excess of diagnosable mental health disorders was also found among Ukrainian liquidators compared to controls 18 years later (Loganovsky *et al*. 2008).

The Fukushima nuclear power plant workers were exposed to multiple stressors at the facility, such as repeated earthquakes and tsunamis, plant explosions, and loss of colleagues. A large majority of them were local residents and thus suffered victim experience (e.g. home evacuation) or loss of family members. Some of them have also been subjected to discrimination and slurs (*sabetsu* and *chuushou* in Japanese) stemming from widespread criticism of TEPCO's post-disaster management. A cross-sectional study, conducted 2–3 month post-disaster, revealed that 29.5% of Daiichi workers *v.* 19.2% of Daini workers had post-traumatic stress responses (PTSR), and that exposure to discrimination/slurs was the most significant correlate of both groups' mental health (Shigemura *et al*. 2012*b*). The longitudinal mental health effect of these workers, however, is yet to be reported.

The objective of this follow-up study is to examine whether discrimination/slurs had a sustained influence on the groups' mental health 1 year later. To our knowledge, this is the first study to longitudinally describe the mental health of a representative sample of Fukushima nuclear power plant workers.

## Method

### Participants

This study was conducted as a part of Fukushima NEWS Project (NEWS; Nuclear Energy Workers' Support), a longitudinal study of Daiichi and Daini workers (Shigemura *et al.* 2012*b*, 2014). Following approval by the Ethics Committees of Ehime University and National Defense Medical College, we recruited full-time employees of TEPCO who were working at Daiichi and Daini when the Fukushima accident occurred.

**Fig. 1.**
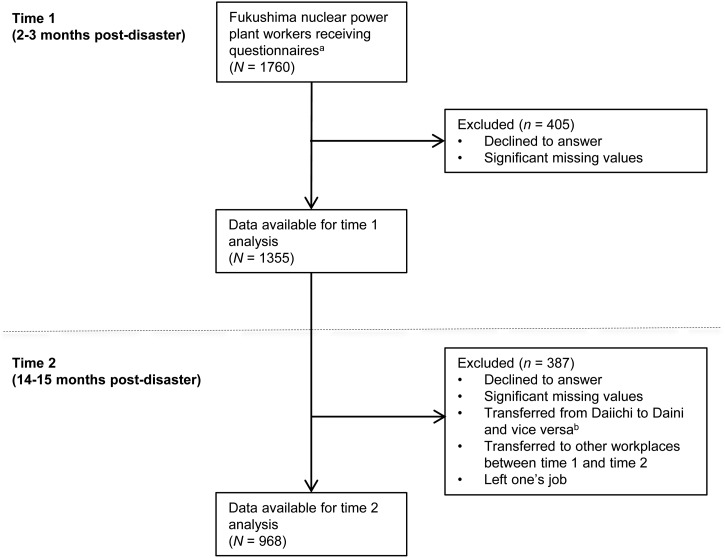
Flow chart of the recruitment process. ^a^ Full-time workers of Fukushima Daiichi and Daini nuclear power plants. ^b^ Workers who transferred from Daiichi to Daini (37 workers) and vice versa (46 workers) after time 1.

Fig. 1 shows flow chart of the recruitment process. At time 1 (2–3 months post-disaster, May–June 2011), questionnaires were given to 1760 workers (Daiichi, *n* = 1053; Daini, *n* = 707). Of those who received questionnaires, 405 workers were excluded due to failure to sign the consent form or returning incomplete questionnaires. Thus, time 1 data were available for 1355 respondents.

At time 2 (14–15 months post-disaster, May–June 2012), we re-recruited the original sample of TEPCO workers who continued to work at either the Daiichi or Daini nuclear power plants. Of the original 1355 workers, a total of 387 respondents did not sign the follow-up consent form, returned incomplete questionnaires, transferred from Daiichi to Daini and vice versa, transferred elsewhere after time 1, or left their job after time 1. Thus, the final number of respondents included in the analysis was 968 (final response rate 55.0%; Daiichi, *n* = 571, 54.2%; Daini, *n* = 397, 56.2%).

### Outcomes and measures

We used self-report questionnaires to assess mental health status at time 1 and time 2. The time 1 questionnaires included demographic characteristics and disaster-related stressors (coded dichotomously as ‘yes’ or ‘no’). The disaster-related stressors were: discrimination/slurs, experience of life-threatening danger [referred to in our previous study as ‘near death’ (Shigemura *et al.* 2012*b*, 2014)], escape from tsunami, witness of plant explosion(s), family member death(s), colleague death(s), major property loss, home evacuation, and peritraumatic distress (PD) as measured by the Japanese version of Peritraumatic Distress Inventory (PDI). The PDI is a 13-item scale with larger numbers indicating larger psychological distress at the time of or immediately after an individual's traumatic exposure (Nishi *et al.* 2010).

General psychological distress (GPD) and PTSR were evaluated at both time 1 and time 2, and these variables at time 2 were our outcome variables. GPD was assessed using the Japanese version of the K6, a 6-item scale including questions on feeling ‘nervous’, ‘hopeless’, ‘restless/fidgety’, ‘so depressed that nothing could cheer you up’, ‘everything was an effort’, and ‘worthless’ in the preceding 30 days (Kessler *et al.* 2003; Furukawa *et al*. 2008). Scores ranged from 0 to 24. The scale has good internal consistency (Cronbach's α: time 1, 0.88; time 2, 0.89).

PTSR was assessed using the Japanese version of the Impact of Event Scale – Revised (IES-R), a 22-item scale covering DSM-IV post-traumatic stress disorder (PTSD) domains of intrusion, avoidance/numbing, and hyperarousal (Weiss & Marmar, 1997; Asukai *et al*. 2002). Scores ranged from 0 to 88. The scale reliability was excellent (Cronbach's α: time 1, 0.95; time 2, 0.95). PTSR refers to psychological responses of the aforementioned three domains that arise in affected individuals after a traumatic event. On the other hand, PTSD includes the three PTSR domains plus impairment and duration of at least 1 month (APA, 1994).

### Statistical analysis

Demographic information and disaster-related stressors were summarized using means and standard deviations for continuous variables and percentages for dichotomous variables. We calculated the differences of time 1 variables between the study group *v.* the non-follow-up group (*n* = 269) to test the effect of attrition between time 1 and time 2. We conducted two-way analysis of variance (ANOVA) with Bonferroni correction to examine the effect of time (time 1 *v.* time 2), nuclear power plant affiliation (Daiichi *v.* Daini), and time × nuclear power plant site interaction to GPD as well as PTSR at time 2.

We used hierarchical multiple regression analyses to identify independent predictors of GPD and PTSR at time 2. In order to assess categorical variables in the analyses, we created dummy variables ranging from 0 to 1. The analyses process consisted of four models. At model 1, sociodemographic variables at time 1 were entered (i.e. age, gender, and pre-existing illness). For model 2, work status values at time 1 were entered (i.e. nuclear power plant affiliation, supervisory status). Regarding model 3, the following set of disaster-related experience variables at time 1 were entered: discrimination/slurs, experience of life-threatening danger, escape from tsunami, witness of plant explosion(s), family member death(s), colleague death(s), major property loss, and home evacuation experiences. For model 4, we added mental health measures. For GPD at time 2, PD and GPD at time 1 were entered whereas for PTSR at time 2, PD and PTSR at time 1 were entered. In model 4, we excluded PTSR at time 1 for the former and GPD at time 2 for the latter because high correlation between GPD and PTSR posed multicollinearity issues (1 < variance inflation factor < 5; GPD at time 1 *v.* PTSR at time 1: *r* = 0.79, *p* < 0.001; GPD at time 2 *v.* PTSR at time 2: *r* = 0.66, *p* < 0.001).

IBM SPSS Statistics v. 22 (IBM Corp., USA) was used for the analysis. Two-tailed significance was set at *p* < 0.05.

## Results

Table 1 shows the workers' demographic characteristics and disaster-related experiences at time 1. The mean age at time 1 was approximately 40 years, and more than 90% of the workers were male. About 60% of the workers were affiliated with the Daiichi plant. The workers had various disaster-related experiences. Regarding the attrition effect, the study group was older (study group *v.* non-follow-up group: 39.8 ± 11.2 *v.* 38.1 ± 10.8; *t* = −2.23, *p* = 0.026), had a lower percent with supervisory status (9.5% *v.* 14.1%, χ^2^ = 4.78, *p* = 0.029), was less likely to report life-threatening danger (39.3% *v.* 50.6%, χ^2^ = 11.1, *p* = 0.001), and was more likely to report home evacuation (69.2% *v.* 56.1%, χ^2^ = 16.1, *p* < 0.001).

**Table 1.** Characteristics of demographics and experiences of the participants at time 1

	Total (*N* = 968)	
	*n*	%
Demographics		
Age (years)	39.8 (11.2)^a^^,^^b^	
Sex (male)	909	93.9
Pre-existing illness(es)	139	14.4
Nuclear power plant (Daiichi)^c^	571	59.0
Supervisory work status	92	9.5
Disaster-related experiences		
Discrimination and slurs	131	13.5
Experience of life-threatening danger	380	39.3
Escape from tsunami	122	12.6
Witnessing plant explosion(s)	257	26.5
Family member death(s)	57	5.9
Colleague death(s)	168	17.4
Major property loss	288	29.8
Home evacuation	670	69.2
Peritraumatic Distress Inventory	17.96 (9.20)^a^	

Time 1, 2–3 months post-disaster.

a Mean (standard deviation).

b Age at time 1.

c Daiichi nuclear power plant suffered meltdown damage. Other participants are from Daini (nearby plant, without meltdown damage).

Table 2 displays the relationships between GPD and PTSR, time, and nuclear power plant site. The test for the main effect of time showed a significant chronological decline in the levels of GPD and PTSR (GPD: *F* = 221.8, *p* < 0.001; PTSR: *F* = 194.0, *p* < 0.001). The test for the main effect of nuclear power plant revealed the site differences of GPD and PTSR (GPD: *F* = 12.9, *p* < 0.001; PTSR: *F* = 23.8, *p* < 0.001). An interaction effect of time × nuclear power plant site was found both for GPD and PTSR (GPD: *F* = 10.0, *p* = 0.002; PTSR: *F* = 11.2, *p* = 0.001).

**Table 2.** General psychological distress, posttraumatic stress responses, and their interactions with time and site (N = 968)

	K6				IES-R			
	df	Mean square	*F*	*p*	df	Mean square	*F*	*p*
Mean (standard deviation)								
Time 1	6.39 (5.12)^a^				17.20 (15.49)^b^			
Time 2	4.02 (4.01)				11.13 (12.04)			
Two-way ANOVA with interaction								
Time^c^	1	2425.9	221.8	<0.001***	1	15 821.0	194.0	<0.001***
Nuclear power plant^d^	1	400.0	12.9	<0.001***	1	7030.8	23.8	<0.001***
Time × nuclear power plant	1	109.0	10.0	0.002**	1	913.3	11.2	0.001**

Time 1, 2–3 months post-disaster; time 2, 14–15 months post-disaster; K6, K6 scale; IES-R, the Impact of Event Scale – Revised, ANOVA, analysis of variance.

a Correlation between Peritraumatic Distress Inventory (time 1) and K6 (time 1): *r* = 0.68, *p* < 0.001.

b Correlation between Peritraumatic Distress Inventory (time 1) and IES-R (time 1): *r* = 0.68, *p* < 0.001.

c Time: Comparison between time 1 and time 2.

d Nuclear power plant: Comparison between Fukushima Daiichi (meltdown) and Daini (no meltdown) nuclear power plants.

**p < 0.01, ***p < 0.001.

Table 3 represents the hierarchal multiple regression analysis of higher GPD at time 2. Model 4 explained 24.1% of the model variance. In this model, the predictors of higher GPD at time 2 were discrimination/slurs experiences at time 1 (standardized *β* = 0.065, *p* = 0.025) as well as higher GPD at time 1 (standardized *β* = 0.491, *p* < 0.001).

**Table 3.** Prospective relationship between independent variables at time 1 and general psychological distress at time 2 (N = 968)

	Time 2 K6 hierarchical models			
	Model 1	Model 2	Model 3	Model 4
Time 1 variables	Adjusted *β*	Adjusted *β*	Adjusted *β*	Adjusted *β*
I. Sociodemographics				
Age	0.045	0.073*	0.070	0.040
Gender^a^	0.072*	0.074*	0.071*	0.018
Pre-existing illness(es)^b^	0.095**	0.095**	0.090**	0.042
II. Work status^b^				
Nuclear power plant^c^		−0.059	−0.028	0.009
Supervisory status^b^		−0.087*	−0.074*	−0.045
III. Disaster-related experiences^b^				
Discrimination and slurs			0.140***	0.065*
Experience of life-threatening danger			0.053	−0.007
Escape from tsunami			0.033	0.008
Witnessing plant explosion(s)			0.015	0.010
Family member death(s)			−0.011	0.012
Colleague death(s)			0.010	−0.014
Major property loss			0.048	−0.001
Home evacuation			0.010	0.001
IV. Mental health measures				
PDI at time 1				−0.031
K6 at time 1				0.491***
Statistics				
Adjusted *R*^2^	0.014**	0.022***	0.048***	0.241***
Δ*R*^2^		0.008	0.026	0.193

PDI, Peritraumatic Distress Inventory; K6, K6 scale; time 1, 2–3 months post-disaster; time 2, 14–15 months post-disaster.

a Dummy variable was created for a categorical variable (male = 0, female = 1).

b Dummy variable was created for a categorical variable (no = 0, yes = 1).

c Dummy variable was created for a categorical variable (Daiichi = 0, Daini = 1).

*p < 0.05, **p < 0.01, ***p < 0.001.

Table 4 shows the hierarchal multiple regression analysis of higher PTSR at time 2. Model 4 explained 35.5% of the model variance. In this model, higher PTSR at time 2 was associated with higher age (standardized *β* = 0.085, *p* = 0.005), discrimination/slurs experiences at time 1 (standardized *β* = 0.079, *p* = 0.003), and higher PTSR at time 1 (standardized *β* = 0.548, *p* < 0.001).

**Table 4.** Prospective relationship between independent variables at time 1 and posttraumatic stress responses at time 2 (N = 968)

	Time 2 IES-R hierarchical models			
	Model 1	Model 2	Model 3	Model 4
Time 1 variables	Adjusted *β*	Adjusted *β*	Adjusted *β*	Adjusted *β*
I. Sociodemographics				
Age	0.079*	0.106**	0.112**	0.085**
Gender^a^	0.068*	0.074*	0.074*	0.015
Pre-existing illness(es)^b^	0.100**	0.099**	0.092**	0.020
II. Work status^b^				
Nuclear power plant^c^		−0.106**	−0.066*	−0.002
Supervisory status^b^		−0.086*	−0.073*	−0.035
III. Disaster-related experiences^b^				
Discrimination and slurs			0.192***	0.079**
Experience of life-threatening danger			0.045	−0.035
Escape from tsunami			0.065*	0.019
Witnessing plant explosion(s)			0.042	0.031
Family member death(s)			0.037	0.038
Colleague death(s)			0.038	0.008
Major property loss			0.024	−0.023
Home evacuation			0.010	−0.002
IV. Mental health measures				
PDI at time 1				0.024
IES-R at time 1				0.548***
Statistics				
Adjusted *R*^2^	0.020***	0.036***	0.089***	0.355***
Δ*R*^2^		0.016	0.053	0.266

PDI, Peritraumatic Distress Inventory; IES-R, the Impact of Event Scale Revised; time 1, 2–3 months post-disaster; time 2, 14–15 months post-disaster.

a Dummy variable was created for a categorical variable (male = 0, female = 1).

b Dummy variable was created for categorical variables (no = 0, yes = 1).

c Dummy variable was created for a categorical variable (Daiichi = 0, Daini = 1).

*p < 0.05, **p < 0.01, ***p < 0.001.

## Discussion

Our findings indicate the mental health outcomes and their predictors of the Daiichi and the Daini workers 14–15 months after the Fukushima nuclear disaster. To our knowledge, this is the first large-scale longitudinal study to examine the year-long impact of disaster-related stress on mental health among nuclear power plant workers directly affected by the Fukushima nuclear disaster, or any other nuclear disaster of this scale.

Our data revealed GPD at time 2 was predicted by baseline GPD at time 1. Likewise, PTSR at time 2 was predicted by baseline PTSR at time 1. These findings indicated individuals with higher mental health responses at the initial phase have risks of year-long mental health responses. Our findings thus support the need for early mental health screenings among nuclear disaster workers. These efforts, along with interventions, might be essential to identify individuals at risk for future adverse outcomes and to mitigate their responses. Future studies will be needed to test this hypothesis.

Higher GPD and PTSR at time 2 were predicted by discrimination/slurs experience at time 1. Studies have highlighted nuclear accidents as one of the most important challenges in emergency management, owing to the uncertainty and fear among the public (Perko, 2011). In nuclear crisis communications, one of the main public health goals is to prevent fear-driven public response (Perko, 2011). When this goal not achieved, however, public responses might lead to discrimination, stigmatization, and scapegoating of certain populations (Glik, 2007). Following the Fukushima nuclear disaster, the public heavily criticized TEPCO for their post-disaster management decisions. Among the Fukushima nuclear power plant workers at both the affected (Daiichi) and unaffected (Daini) sites, discrimination and stigmatization became an issue that affected their post-disaster everyday lives (Shigemura *et al.* 2012*a*). Our result extends our previous cross-sectional report (Shigemura *et al.* 2012*b*) and suggest that negative public responses have a sustained mental health impact on these workers.

In this study, PTSR at time 2 was predicted by older age. This suggests that older workers are more prone to develop PTSR than younger workers. To our knowledge, there is only one study finding regarding the relationship between age and mental health among nuclear power plant workers; a mental health study among TMI workers showing that age was not a contributing factor for their post-disaster mental health (Parkinson & Bromet, 1983). It suggests that our study shows novel findings and importance of support to older workers, but further investigation is needed for more in-depth analysis.

PD at time 1 was not significantly associated with either GPD or PTSR at time 2. There is compelling evidence in the literature showing that emotional distress during a traumatic event is a strong predictor of objective severity of the event. This association was reported following a natural disaster (Cénat & Derivois, 2014) and in armed conflict survivors (Agorastos *et al.* 2013) as well as in first responders such as police officers (Marmar *et al.* 2006) and rescue workers (Nishi *et al.* 2012). In our time 1 cross-sectional report (Shigemura *et al*. 2014), PD mediated the association between traumatic exposure and PTSR. In this report, however, we were unable to show a definite relationship between PD and mental health responses at time 2, and this result might be due to high correlations between time 1 PD, GPD, and PTSR. We do not know of any other longitudinal studies assessing this relationship among the nuclear power plant workers immediately following a disaster. Additional studies are needed to understand the role of PD and subsequent mental health responses among this population.

### Strengths and limitations

There are several study limitations that must be considered in evaluating the results of the current study. First, the sample consists of employees from a single company, but there are several companies that staff the Fukushima nuclear power plants. Thus, our study findings do not represent all of the Fukushima nuclear power plant workers. Second, this study was assessed using self-report questionnaires rather than interviews. Third, we did not assess some demographic variables (e.g. marital status, income, presence or absence of children, detailed job descriptions) that might also have been associated with the psychological outcomes. Fourth, workers who were not in the follow-up had different demographics than the study group, such as higher rate of life-threatening experience. We do not know the reasons of non-follow-up at time 2, and caution should be exercised in generalizing from the results. Fifth, given that approximately 1 year had passed since the disaster exposure when the follow-up questionnaires were administered, other potential traumatic events may have contributed to the outcomes. Last, there is little evidence about the physical and/or mental health influence of their radiation exposure. There has been no acute radiation syndrome or any other serious radiation-related health crisis to the Fukushima nuclear power plant workers during this time period (Hiraoka *et al.* 2015; Shimura *et al.* 2015). Still, more potential predictors should be included in future studies of nuclear power plant workers after major meltdowns as happened in Fukushima.

The current study also has a number of offsetting strengths, including a unique sample as well as the 1-year follow-up design. To our knowledge, this is the first study to clarify the temporal association between discrimination/slurs and mental health status among nuclear power plant workers following a large-scale nuclear disaster.

## Conclusion

Among the Fukushima nuclear power plant workers post-nuclear disaster, the predictors of higher GPD at time 2 were: higher GPD at time 1 and discrimination/slurs experience at time 1. The predictors of higher PTSR at time 2 were: higher PTSR at time 1, discrimination/slurs experience at time 1, and older age. Early mental health screenings and interventions might be helpful to identify at-risk individuals and to provide intervention programmes. Considering findings that discrimination/slurs significantly influence the long-term mental health of these workers, strategies to reduce public criticism are likely to reduce emergence of negative mental health outcomes. Careful mental health support for older workers might also need to be considered. Further investigation is essential for more insightful analysis of the long-term mental health of this population.

## Appendix. Fukushima NEWS Project Collaborators

Shoichi Tachibana, M.D., Ph.D.^1,2^, Shin-ya Sano, M.D., Ph.D.^3^, Chiyo Fujii, M.D., Ph.D.^4^, Tatsuro Kuwahara, M.D., Ph.D.^5,6^, Yasutaka Tatsuzawa, M.D., Ph.D.^5^, Yutaka Sato, MA^5^, Hiroyuki Toda, M.D., Ph.D.^5^

^1^*Division of Environmental Medicine, Defense Medicine Research Institute, National Defense Medical College, Tokorozawa, Saitama Japan*

^2^*Hirasawa Memorial Hospital, Tokorozawa, Saitama, Japan*

^3^*Department of Psychology, National Defense Medical College, Tokorozawa, Saitama Japan*

^4^*Department of Psychiatric Rehabilitation, National Institute of Mental Health, National Center of Neurology and Psychiatry, Kodaira, Tokyo Japan*

^5^*Department of Psychiatry, National Defense Medical College, Tokorozawa, Saitama Japan*

^6^*Department of Psychiatry, Tachikawa Hospital, Tachikawa, Tokyo Japan*
